# Bond Properties of Steel Bar in Polyoxymethylene-Fiber-Reinforced Coral Aggregate Concrete

**DOI:** 10.3390/polym17212954

**Published:** 2025-11-06

**Authors:** Zhuolin Xie, Lin Chen, Lepeng Huang, Junlong Jin, Jianmin Hua, Pow-Seng Yap, Yi Zhang

**Affiliations:** 1 School of Civil Engineering, Chongqing University, Chongqing 400045, China; 2 Key Laboratory of New Technology for Construction of Cities in Mountain Area, Ministry of Education, Chongqing University, Chongqing 400045, China; 3 School of Engineering, Faculty of Innovation and Technology, Taylor’s University, Subang Jaya 47500, Selangor, Malaysia; 4 Centre for Sustainable Societies, Taylor’s University, Subang Jaya 47500, Selangor, Malaysia; 5 Chongqing Construction Residential Engineering Co., Ltd., Chongqing 400015, China

**Keywords:** coral aggregate concrete, polyoxymethylene fiber, fiber reinforcement, drawing test, bond property, destruction pattern, bond–slip constitutive model, anchorage length

## Abstract

The rapid expansion of island and reef infrastructure has intensified the demand for sustainable concrete materials, yet the scarcity of conventional aggregates and freshwater severely constrains their supply. More critically, the fundamental bonding mechanism between steel reinforcement and coral aggregate concrete (CAC) remains poorly understood due to the highly porous, ion-rich nature of coral aggregates and the complex interfacial reactions at the steel–cement–coral interface. Moreover, the synergistic effect of polyoxymethylene (POM) fibers in modifying this interfacial behavior has not yet been systematically quantified. To fill this research gap, this study develops a C40-grade POM-fiber-reinforced CAC and investigates the composition–property relationship governing its bond performance with steel bars. A comprehensive series of pull-out tests was conducted to examine the effects of POM fiber dosage (0, 0.2%, 0.4%, 0.6%, 0.8%, and 1.0%), protective layer thickness (32, 48, and 67 mm), bar type, and anchorage length (2 *d*, 3 *d*, 5 *d*, and 6 *d*) on the interfacial bond behavior. Results reveal that a 0.6% POM fiber addition optimally enhanced the peak bond stress and restrained radial cracking, indicating a strong fiber-bridging contribution at the micro-interface. A constitutive bond–slip model incorporating the effects of fiber content and c/d ratio was established and experimentally validated. The findings elucidate the multiscale coupling mechanism among coral aggregate, POM fiber, and steel reinforcement, providing theoretical and practical guidance for the design of durable, low-carbon marine concrete structures.

## 1. Introduction

In recent years, countries around the globe have invested in ocean engineering to build maritime power, fully exploit marine resources, and protect their territorial sovereignty. Common examples of maritime structures include artificial islands and reefs, airports, docks, and breakwaters [1,2]. However, being far from the mainland, supplying sand, stone, and fresh water is difficult, with the transportation cost from the mainland being high. To support the sustainable development of islands and reefs and align with the global demand for low-carbon and environmentally friendly infrastructure, local materials should be used to produce concrete [3], for instance, by replacing sand and stone in ordinary concrete with sea sand and coral debris [4,5,6,7,8]. This not only lowers costs but also makes full use of the ground material.

Coral reefs are formed by the heavy accumulation of coral bones over hundreds of years of growth and mainly comprise CaCO_3_ [9,10]. Coral reefs are decomposed into coral fragments under the action of sea waves and sea breezes, and these weathered and decomposed coral aggregates can be used to replace concrete aggregates. They have extremely high economic significance and important engineering value for island construction [11,12,13]. The 2024 “Xianbin Reef Coral Reef Ecosystem Survey Report” [14] by China’s Ministry of Natural Resources points out that under hydrodynamic forces, these coral fragments can directly bury live corals and cause physical damage such as impact and friction to corals around the buried area. At the same time, they hinder the attachment and growth of coral larvae, leading to damage or death of surrounding corals. Therefore, recycling and reusing coral debris generated in coral reef areas can not only prevent its accumulation as marine waste and reduce pressure on the surrounding ecological environment, but also decrease reliance on limited natural aggregates. This method does not require the extraction of new natural aggregates, thereby reducing energy consumption and carbon emissions during quarry construction, mining, and transportation processes, which aligns with the principles of circular economy and sustainable development.

Indeed, the application of this sustainability principle extends beyond coral debris, with the recycling of other marine by-products also gaining significant research attention. The recycling of seafood and aquaculture waste, such as oyster, mussel, clam, and snail shells, into concrete has become a significant research focus in sustainable construction. Numerous studies have demonstrated that these wastes, rich in calcium carbonate, can partially replace cement or aggregates in concrete, reducing environmental impact and promoting circular economy practices. Properly processed shells (e.g., through cleaning, crushing, and sometimes calcination) have been shown to improve certain properties of concrete, such as workability and durability, when used at optimal replacement levels [15,16]. For example, mussel and oyster shells have been successfully incorporated into both structural and non-structural concrete, as well as in artificial reefs, enhancing ecological performance and providing new habitats for marine life [17,18]. While the inclusion of shell waste may slightly reduce mechanical strength, especially at higher replacement rates, the resulting materials often meet the requirements for non-structural applications and offer substantial environmental benefits [16,17,19]. While these studies provide valuable context for marine waste recycling, coral aggregate, as the primary focus of this paper, possesses a unique set of properties that warrants specific investigation.

Coral aggregate has the characteristics of high porosity, low accumulation density, large water absorption rate, and low cylinder pressure strength, and is classified as natural light aggregate [20,21,22]. Because of its properties, the primary characteristics of coral aggregate concrete (CAC) differ greatly from those of conventional concrete. According to early studies, the compressive strength of CAC is only between 20 and 30 MPa when the design method of ordinary concrete is used [23,24]. Subsequent optimization improved the strength to 50–80 MPa [25,26]. It has been reported that the early strength of CAC increases more rapidly than its later strength due to the presence of chloride ions in the concrete [27,28,29]. Because of the large pore diameter and strong water absorption capacity of the coral aggregate, the cement slurry near the coral aggregate is fully hydrated to have a denser interface transition zone than ordinary concrete during concrete curing, which also improves some of the mechanical characteristics of the CAC [30,31].

The concrete prepared from aggregates obtained from the crushing treatment of coral reefs negatively affects the durability performance of its bonding property with reinforcing bars. This has been attributed to the attachment of harmful ions, such as chloride salts, magnesium salts, and sulfates, in seawater; the deposition of sediment bound by seawater scouring; and the presence of microorganisms on the surface. Meanwhile, seawater is often used in CAC. CAC structures are seldom reinforced directly with ordinary steel bars because of the severe corrosion that would result from the action of seawater, which severely limits the application of CAC [31,32,33,34]; thus, it is only used in non-reinforced concrete structures for constructing islands and reefs. Desalination technology has been used in marine development [35], and seawater quality can match that of drinking water. Fresh water can thus be used to mix CAC on islands and reefs, and reinforced CAC will greatly expand its application in exploiting marine resources.

The bonding property has an important effect on the performance, durability, and bearing capacity of reinforced concrete structures. The material properties of concrete crucially affect its bonding characteristics; specifically, the brittleness and low tensile strength of concrete restrict the bonding strength. Currently, different types of fibers are being mixed into concrete to improve its mechanical properties [11,30,32]. Fiber materials can greatly improve the brittleness and toughness of CAC, significantly enhance its flexural performance, change its failure form, and ensure integrity is maintained even when the specimen is damaged [36]. Although a reasonable fiber content can enhance the compressive, tensile, and flexural strength of CAC, more can be achieved by using the optimal fiber content [37]. In addition, increasing the fiber content gradually decreases the workability of CAC, and the overall slump value exhibits a downward trend [38].

The addition of fiber influences the mechanical characteristics of concrete and its bonding capabilities with reinforced concrete to varying degrees. The failure mode of the reinforced concrete drawing specimen is altered by the addition of fiber. Within a certain range of fiber content, the ultimate bond stress between steel bars and concrete increases with the fiber content [39]. An appropriate fiber content promotes the bridging effect of fibers, improving the ductility, effectively limiting the formation of radial cracks in the reinforcing bars, and effectively transferring the bond stress between the reinforcement bars and concrete [40]. Moreover, fibers can improve the fracture energy of concrete bonding mechanics [41], thus improving the bonding property of reinforced concrete. Studies on the bonding property of alkali-resistant glass fiber-reinforced CAC and basalt fiber-reinforced plastics bars reveal that the maximum mean bond stress and residual friction stress generally decrease with increasing bonding length, and different types of alkali-resistant glass fiber have different improvement effects on the bonding strength [42]. The influence mechanism of fibers on the bonding property of steel bars and CAC is complicated. It is more likely to pull out damage when the right amount of fiber is added. As the amount of incorporated fibers increases, the bonding strength of the deformed steel bar first increases and then decreases; however, the fiber content does not significantly affect the bonding strength of the round-steel-bar specimen.

To systematically compare the effects of different fiber types discussed in the literature and to provide a broader context for the materials used in this study, the performance impacts of various common polymer and other fibers on concrete are summarized in Table 1.

Polyoxymethylene (POM) fiber is a high-performance organic synthetic fiber with excellent comprehensive performance: high strength and high elastic modulus, excellent wear resistance, alkali resistance, high elongation recovery, scratch and fracture resistance, and long-term stability [54]. Its molecular structure comprises a large number of ether bonds. It has good compatibility with inorganic materials and high binding strength, and can be used for improving the cracking resistance of cement-based materials. The enhanced concrete has good homogeneity and mechanical properties, which are better than those achieved using popular fibers, such as polypropylene. Studies of the bonding property of POM-fiber-reinforced concrete reveal that the bond failure modes of POM fiber seawater sea-sand concrete and reinforcement are split failure and pull-out failure. While fiber can improve the peak bond stress, POM fiber deteriorates the bond performance [55]. At present, reinforcing CAC by adding POM fiber has not been sufficiently studied, and no relevant research on the bond performance of POM-fiber-reinforced CAC exists.

In summary, previous studies have primarily focused on the mechanical and durability behavior of CAC or fiber-reinforced CAC, but the interfacial bonding mechanism between steel reinforcement and POM-fiber-reinforced CAC has not been quantitatively clarified. Existing research also lacks a unified model to describe how the coupled effects of fiber content and concrete protective layer thickness influence the bond–slip relationship. Understanding this interfacial mechanism is essential for improving the structural performance and durability of reinforced CAC, especially under the harsh marine environment where the porous coral aggregate and chloride-rich conditions intensify steel–concrete interaction. Incorporating POM fibers offers a potential pathway to enhance the bond strength and crack resistance of CAC, yet its effect has not been systematically investigated. To address this gap, considering the specific conditions of island and reef construction, this study utilizes locally sourced coral aggregates to replace conventional coarse aggregates and develops a C40-grade POM-fiber-reinforced CAC to investigate its bonding performance with steel bars. Through a series of pull-out tests incorporating different fiber dosages, protective layer thick-nesses, bar surface morphologies, and anchorage lengths, the stress development and bond failure modes of reinforced specimens are systematically analyzed. By revealing the multiscale coupling mechanism at the steel–cement–coral interface modified by POM fibers and establishing a validated constitutive bond–slip model linking micro-structural interaction with macroscopic performance, this work provides new insight into the bond behavior of reinforced CAC. The proposed model and anchorage design recommendations form a theoretical and technical foundation for the broader application of CAC in marine engineering, addressing key challenges such as resource recycling, carbon footprint reduction, and material performance enhancement to promote the sustainable development of island and reef infrastructure.

## 2. Experiment Design

**Figure 1:**
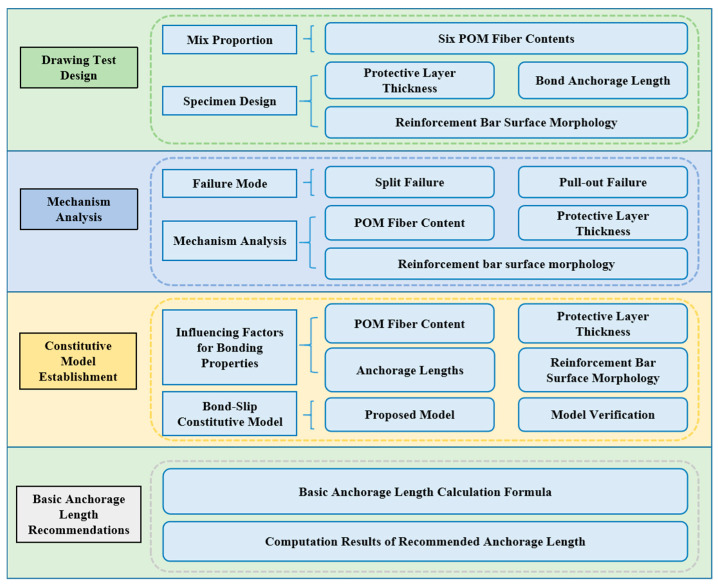
Schematic diagram of bond behavior analysis for POM-fiber-reinforced CAC.

This study designed drawing tests to investigate the bond behavior between steel bars and POM-fiber-reinforced CAC. The failure modes and mechanisms were analyzed, factors influencing bond performance were examined, a bond–slip constitutive model was established, and recommended values for the basic anchorage length were proposed. Figure 1 illustrates a specific flow chart of the study.

### 2.1. Material

#### 2.1.1. POM Fiber

**Figure 2:**
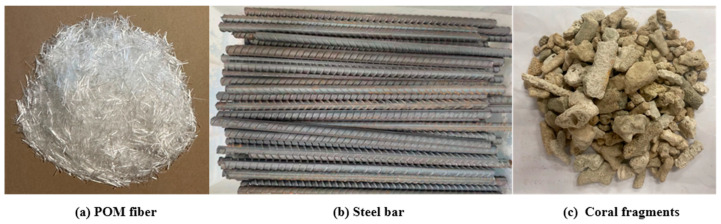
The material of CAC and steel bar: (**a**) POM fiber; (**b**) Steel bar; (**c**) Coral fragments.

The POM fiber used in the experiment was manufactured by Yuntianhua Co., Ltd., Kunming, Yunnan, China. Table 2 presents its principal characteristics. The diameter of the POM fiber is 0.2 mm, and its length is 12 mm, as shown in Figure 2a.

#### 2.1.2. Steel Bar

For the test, a HRB400-class hot-rolled deformed reinforcement bar with a diameter of 16 mm and HPB300 hot-rolled round reinforcement bar with a diameter of 16 mm were used, as shown in Figure 2b. Their basic mechanical property parameters are listed in Table 3.

#### 2.1.3. Concrete Raw Materials and Mix Proportion

Untreated coral aggregate obtained by crushing and size screening coral debris was used in this experiment, as shown in Figure 2c. The proportion of the mass of particles with a diameter of 4.75–9.5 mm and 9.5–16 mm was 45% and 55%, respectively, and the distribution of coral aggregate was in line with that of 5–16 mm aggregate in Chinese standard GB/T17431.1-2010 [56]. The experiment used P.O. 42.5 ordinary silicate cement produced by Esheng Cement Factory in Emeishan City, Sichuan Province, and the parameter indicators of this cement are shown in Table 4. Other cementing materials were grade 1 fly ash and grade S95 mineral powder, as listed in Table 5 and Table 6. In addition, river sand with a fineness modulus of 2.7 and clay content of 0–1% was used as fine aggregate. In this test, the target strength of the concrete was C40, the water-cement ratio (W/B) was 0.25, and the aggregates had been pre-wetted before the concrete was mixed. The concrete was mixed with tap water, using the polycarboxylate type high-efficiency water reducer used in Ultra-High-Performance Concrete (UHPC) as the admixture. The mixing time was 5 min. The blending ratios and working performances of CAC of different fiber contents are listed in Table 7. All the concrete samples were cured for 28 days under standard conditions (room temperature 20 ± 2 °C, relative humidity not less than 95%). A 2000 kN electrohydraulic servo press was used to conduct mechanical tests on 100 × 100 × 100 mm and 150 × 150 × 300 mm concrete test blocks from the same batch with similar physical performances, as listed in Table 8. CAC denotes ordinary coral concrete. POM denotes POM-fiber-reinforced CAC, and the number after POM denotes the fiber content percentage.

### 2.2. Drawing Specimen Design

**Figure 3:**
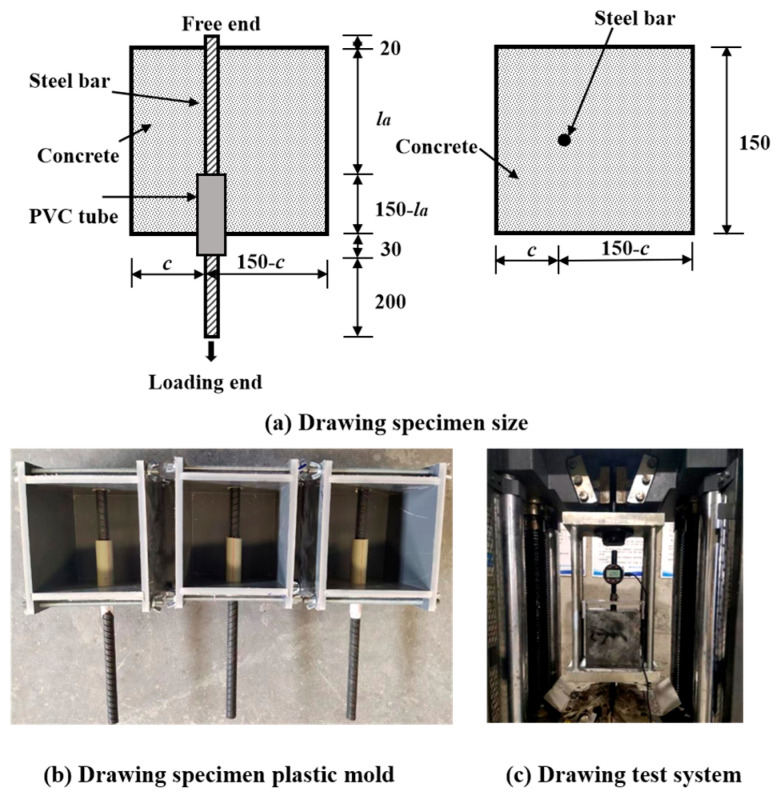
Drawing specimen and drawing test system: (**a**) Drawing specimen size; (**b**) Drawing specimen plastic mold; (**c**) Drawing test system.

In accordance with Chinese standard GB50152-92 [57] and GB/T50081-2019 [58], the concrete specimens were 150 × 150 × 150 mm. The two ends of the steel bar in the specimen are the loading end and the free end to facilitate pull loading and measurement of free-end displacement. Figure 3a illustrates the exact specimen dimensions. The bond stress was evenly distributed over a short anchorage length. Furthermore, the bond anchorage length was adjusted by embedding PVC sleeves of different lengths at the loading end to eliminate the adverse effects of local stress concentration at the loading end and in the concrete during the test, as shown in Figure 3b. Glass glue is required on one side after inserting the steel bar into the PVC pipe sleeve to prevent concrete leakage during pouring and vibration. The PVC sleeve inside was sealed with foam glue, and the free end of the steel bar was fixed and sealed with packaging tape to prevent concrete leakage during pouring and vibration.

The main factors considered in this experiment were as follows: (1) the influence of POM fiber content, namely the content of undoped fiber, 0.2%, 0.4%, 0.6%, 0.8%, and 1% fiber; (2) protective layer thickness (*c*): 32 mm, 48 mm, and 67 mm (center drawing); (3) bond anchorage length (*la*): for the reinforcement bar with a diameter of 16 mm, bond lengths of 4 times, 5 times, and 6 times the reinforcement bar diameter were set; (4) reinforcement bar surface morphology: deformed (D) and smooth-surface (S).

For this test, 27 sets of specimens were made, with 3 specimens from each group, for a total of 81 drawing specimens. The specimen groupings and their different parameters are listed in Table 9 (C represents CAC, POM represents POM-fiber-reinforced CAC, and the value after POM represents the POM fiber content. The values before D and S indicate that the anchorage length is a multiple of the reinforcement bar diameter. Finally, 2, 3, and 4.2 in the third part of the number represent the ratio of the protective layer thickness to the reinforcement bar diameter.

### 2.3. Loading Device and Loading System

A pull-out test was conducted to evaluate the bonding performance. The loading device in this test mainly comprised a 600 kN microcomputer controlled electrohydraulic servo universal testing machine and a rigid reaction hanger. The loading hanger was composed of two steel plates with a central drilling hole approximately 30 mm thick and 5 high-strength screws, as shown in Figure 3c. A dial gauge was installed at the free end of the steel bar, with its clamp fixed to the concrete surface, thus recording the relative slippage between the free end of the steel bar and the concrete.

To ensure precision in the test, the drawing test was conducted under a slow and steady load control mode. The loading velocity was calculated to match the diameter of the reinforcement bar. With reference to relevant standards [57,58], Equation (1) is used to calculate the loading speed:

*V =* 0.03 *d*^2^ (1)

where *V* denotes the loading speed (kN/min) and *d* denotes the reinforcement diameter (mm).

Accordingly, the loading control speed of the 16 mm-diameter steel bar drawing specimen was calculated to be 150 N/s.

Before actual loading, preloading to 2 kN was applied to ensure the specimen was in full contact with the testing machine, after which it was unloaded to 0 kN and actual loading applied. A stable loading rate was applied until specimen failure. During the testing, the rebar slippage at the free end was recorded by a dial gauge setup, while the load F was automatically acquired by the load-testing machine. All test data were collected at a sampling frequency of 10 Hz. From the test, the bond stress can be calculated using Equation (2):

*τ* = *F*/(*πdl_a_*) (2)

where *F* denotes applied load (N), *d* denotes reinforcement diameter (mm), and *l_a_* denotes the anchorage length (mm).

## 3. Results and Discussion

To enhance the accuracy of the experiment, the average of three specimens per group was adopted for analysis. Key parameters from the pull-out tests—average value of ultimate load, peak bond stress *τ_u_*, peak slip *s_u_*, and failure modes—are compiled in Table 10. The failure forms of the pull-out specimen throughout the test were primarily split failure, pull-out with split failure and pull-out failure.

### 3.1. Failure Mode and Mechanism Analysis

**Figure 4:**
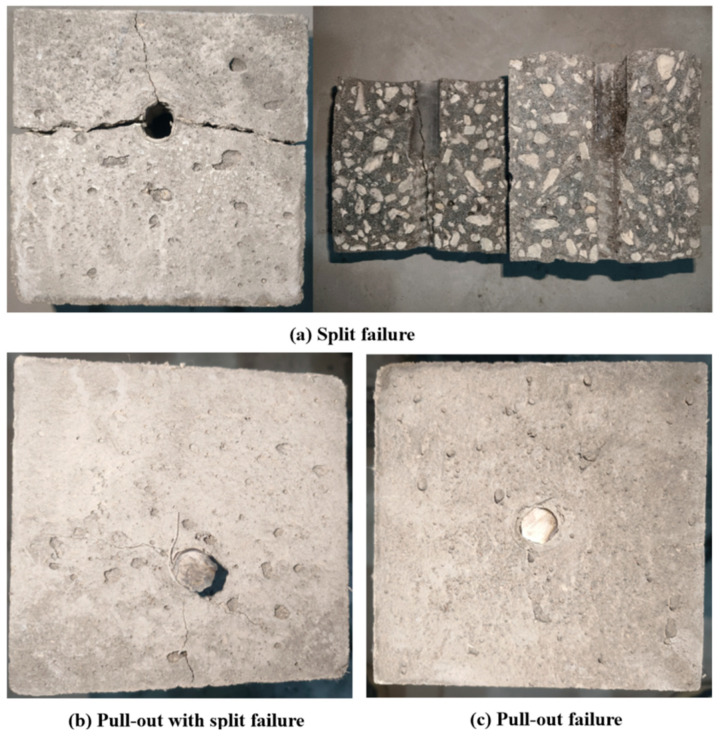
Typical failure modes of deformed-bar pulling specimens: (**a**) Split failure; (**b**) Pull-out with split failure; (**c**) Pull-out failure.

During the pull-out test, the observed bond failure modes include split, pull-out with split, and pull-out, which represent the interfacial bond failure mechanisms between deformed steel bars and CAC. Some specimens failed after the sudden fracture of CAC. No prominent warning preceded failure, which occurred rapidly. At the beginning of loading, the slip amount of the steel bar was very small, but it gradually increased with the increase in the tensile force at the loading end. As the tensile force approached the peak value, cracks appeared in the CAC specimen and developed rapidly with the increase in tensile force. At peak tensile force, the radial force of the rib along the reinforcement exceeded the anti-splitting ability of the concrete test block [34]. Accordingly, the CAC split with a loud sound, and the specimen ultimately broke. The specimen surface developed penetrating cracks and split into two or three fragments, as illustrated in Figure 4a. Some specimens exhibited pull-out with split failure. Although cracks developed, these specimens remained structurally sound without disintegration, featuring narrow crack widths with partially non-penetrating cracks that did not propagate to the specimen edges, as depicted in Figure 4b. When the specimen was pulled out, no crack was on the surface of the specimen, and the entire specimen appeared intact, as shown in Figure 4c. The drawing load gradually overcame the resistance, and the bite teeth in the CAC between the reinforcement ribs were cut. The reinforcement bar was then pulled out along with the concrete fragments filled between the ribs. Ground concrete powder was observed after the bar was pulled out.

The drawing test revealed that the amount of POM fiber has little effect on the failure mechanism for the specimen with the plain round reinforcement bar, and all the plain round bar bond specimens were pulled out because they lacked the mechanical bite force of the steel rib. No splitting cracking occurred on the CAC surface; the surface of the steel bar was devoid of concrete residue. Moreover, a little ground concrete powder was observed after pulling out the reinforcement bar.

As listed in Table 10, the bonding failure mechanism between the deformed steel bar and CAC specimen was dependent on the thickness of the protective layer. The smaller the protective layer thickness, the greater the tendency of the specimen to crack. With the reduction in the protective layer, the circumferential binding force between concrete and the reinforcement bar decreased, and the concrete became more prone to split failure, the amount of circumferential cracks increased, and the crack breadth increased. Notably, POM-fiber-reinforced CAC was prone to pull-out with split failure.

### 3.2. Bond–Slip Curve and Model

#### 3.2.1. Bond–Slip Curve

**Figure 5:**
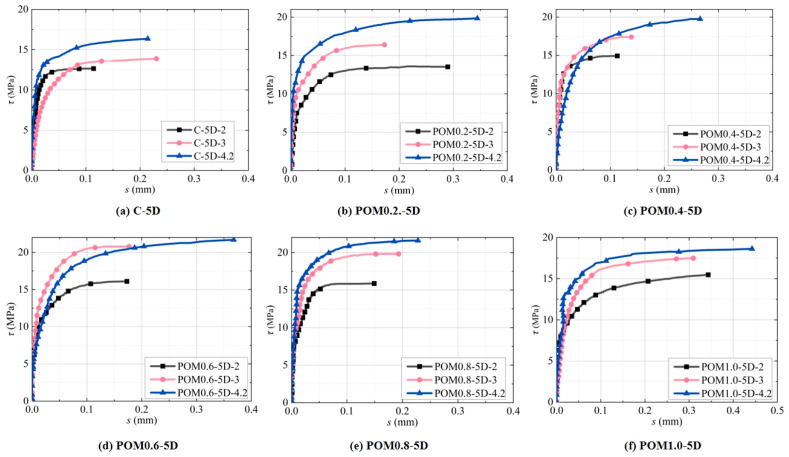
Bond–slip curve of POM-fiber-reinforced coral concrete: (**a**) C-5D; (**b**) POM0.2-5D; (**c**) POM0.4-5D; (**d**) POM0.6-5D; (**e**) POM0.8-5D; (**f**) POM1.0-5D.

According to Figure 5, the bond curve of the 28 d deformed steel bar and POM-fiber-reinforced CAC exhibited three stages:

(1) Microslip stage: At the beginning of the loading stage, the load (*F*) and slip (*s*) of the testing machine exhibited a largely linear relationship, the loading force was small, and the free end exhibited almost no displacement. Meanwhile, the chemical adhesive force between the deformed reinforcement bar and the CAC made up most of the bonding force [59]. The surface of the specimen exhibited no visible distortion or fissures, and the curve was linear.
(2) Slip stage: The curve becomes nonlinear and roughly presents an exponential function form at a peak load ranging between 30% and 100%. With the continuous increase in loading force, the steel rib successively develops cracks from the loading end to the free end, and the bonding force of the specimen was mainly supplied by the mechanical bite force and friction force [60]. The compressive stress in the front rib area increased, resulting in local concrete compression and friction and extrusion between the steel rib and CAC. Moreover, plastic deformation and micro-cracks gradually appeared inside the specimen. By forming a broken zone in front of the reinforcement rib, the radial component of the reinforcement rib pressure on the surrounding concrete creates circumferential tensile stress in the concrete. At this stage, the slip increases rapidly, and the bond–slip curve rises nonlinearly until it reaches a peak point, namely the peak bond stress.
(3) Parallel stage: When the peak bond stress was reached, the slip between the reinforcement and concrete increased, the force remained largely unchanged, and the slip continued to increase. When the circumferential tensile stress exceeded the CAC tensile strength, radial cracks formed inside the specimen and developed from the surface of the steel bar to that of the specimen along the radial direction. Simultaneously, numerous fine cracks emerged on the surface of the concrete specimen. As slip increased, the adhesive force of the specimen was primarily from the friction force between the reinforcement and concrete and the mechanical bite force. Finally, the macroscopic crack developed rapidly, and splitting failure occurred.

The test results for the CAC specimen and POM-fiber-reinforced CAC specimen are summarized in Figure 5. For brevity, only one curve was presented for each test group (classified according to *c*/*d* and fiber content). However, it is not presented in the figure because the descending section after the peak bond stress was reached during splitting failure was extremely irregular, which is considered to have no reference significance. As *c*/*d* increases, the peak bond stress increased correspondingly, and the corresponding slip of the peak bond stress changed marginally. Compared with the CAC specimens, the peak bond stress in the POM-fiber-reinforced CAC was higher, and the gradient of the ascending segment of the curve was steeper. Moreover, the parallel region of the curve after the peak stress was reached was longer. This is because the bending strength and splitting tensile strength of the CAC with added POM fiber improved to a certain extent. The circumferential constraint inside the concrete near the reinforcement bar was greater than that in the CAC, so a greater pulling force was needed to achieve the same degree of damage. In addition, as the protective barrier thickness of concrete increases, the curve’s steepness increases, and the characteristic value *α* of the ascending segment of the curve decreases. This indicates that within the same range, the bond stress increases more rapidly as *c*/*d* increases.

#### 3.2.2. Bond–Slip Constitutive Model

**Figure 6:**
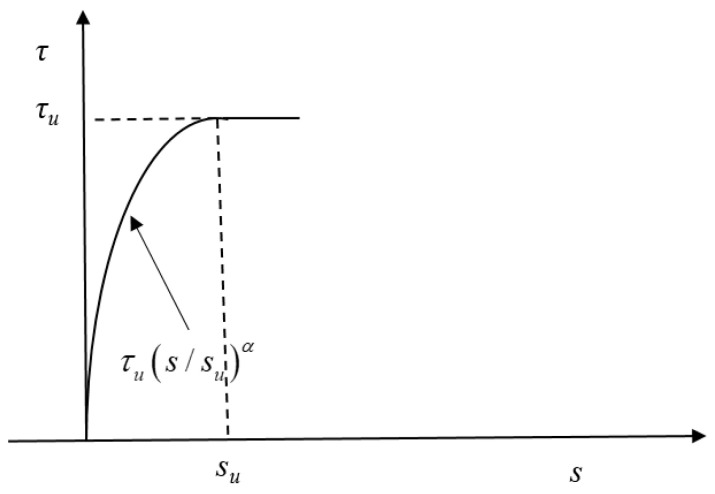
Recommended bond–slip curve [62].

Song et al. [61] developed a mature and classical bond–slip constitutive model. Yang [62] made improvements to the model. In the ascending section, an exponential function was adopted. In the parallel section after reaching the peak stress, the peak bond stress was taken as the characteristic value. Taking into account the shape of the bond–slip curve obtained from the tests, this model can accurately represent the bond behavior between the reinforcing steel and POM-fiber-reinforced coral concrete. Therefore, this model is chosen, as shown in Equation (3), and the schematic diagram of the bond–slip curve is shown in Figure 6.

(3) $$\tau =\left\{\begin{array}{c} \tau_{u}{\left(\frac{s}{s_{u}}\right)}^{\alpha }\;0\leq s\leq s_{u}\\ \tau_{u}\;\;\;s>s_{u} \end{array}\right.$$

where *τ_u_* represents the bond stress, *s* represents slip, *s_u_* represents the slip corresponding to the maximum bond stress, and *α* represents the form parameter of the ascending segment of the bond–slip curve.

The exponential function was applied to the rising section. After the maximum stress was attained, the model was improved according to the experimental results in this section. The peak bond stress had a long parallel section, and the peak bond stress was considered as the characteristic value. According to the specimen curves in this section, no effective falling section in the bond–slip curve of the drawn specimens exists for the splitting failure mode, so the formula was applicable to the rising and parallel sections for the peak bond stress of the specimens under the splitting failure mechanism. The homologous slip *s_u_* to the peak bond stress *τ_u_* and peak bond stress were obtained from the test results. The shape parameter *α* of the rising section was obtained via nonlinear regression analysis based on the least square method in MATLAB (2022b) software. The three curve characteristic values of the specimens were obtained according to the above definition and test results, as presented in Table 11, and the data were the average values of the three experimental groups.

### 3.3. Influencing Factors for Bonding Properties

#### 3.3.1. Influence of Different Fiber Contents

**Figure 7:**
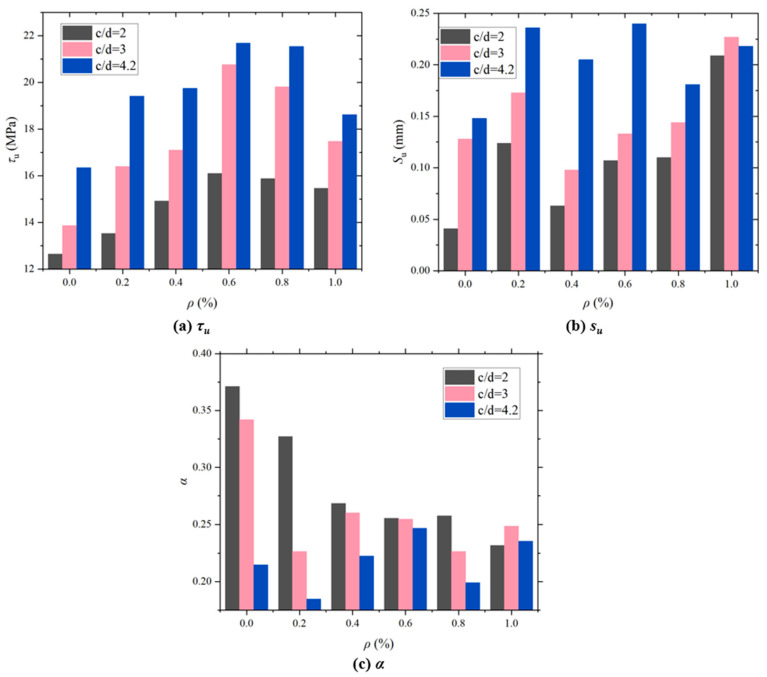
Influence of POM fiber content on the characteristic value of bond–slip curve: (**a**) *τ_u_*; (**b**) *s_u_*; (**c**) *α*.

When *c*/*d* = 2, 3, and 4.2 and the fiber content was 0.6, the peak bond stress *τ_u_* of CAC with distinct POM fiber was 27.24%, 49.69%, and 32.63% higher than those of CAC without fiber, respectively. The peak bond stress of the CAC specimen was highest at a fiber content of 0.6, indicating the best bond performance. As shown in Figure 7, *τ_u_* initially increased and subsequently decreased as the POM fiber content increased. This is because excessive fibers reduce the density of the steel-reinforcing matrix interface, resulting in a decrease in bond strength [63]. When the fiber content is 0.6, the increment of *τ_u_* is the greatest. This is consistent with the research conclusion of carbon fiber-reinforced coral concrete. However, the increment of *τ_u_* in carbon fiber-reinforced coral concrete is the largest at 14.53%, which is smaller than that of POM fibers [32]. The slip *s_u_* corresponding to the maximum stress and form parameter *α* of the rising section were concentrated over a certain interval and have no strong correlation with the POM fiber content.

#### 3.3.2. Impact of Different Protective Layer Thickness

**Figure 8:**
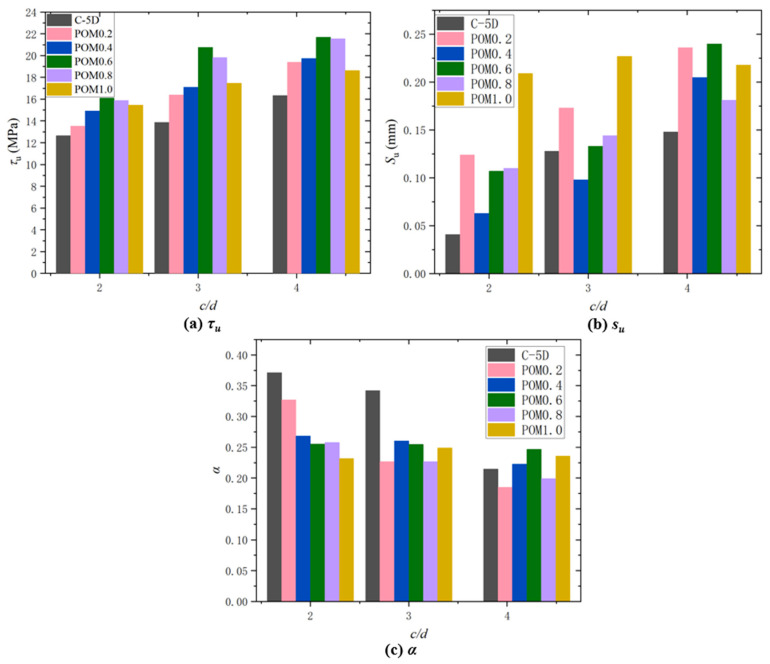
Effect of distinct protective layer thickness on characteristic values of the bond slip curve: (**a**) *τ_u_*; (**b**) *s_u_*; (**c**) *α*.

As shown in Figure 8a, *τ_u_* exhibits a monotonously increasing trend with the increase in concrete relative protective layer thickness *c*/*d*, because increasing the concrete protective layer thickness can enhance the restraining effect of concrete on the reinforcement bar, as well as increase the rigidity of the ascending portion of the bond–slip curve, the peak bond stress, and the residual stress.

Regarding the slip *s_u_* associated with the maximum stress, Figure 8b indicates that with the increase in POM fiber content, *s_u_* concentrated between 0.063 mm and 0.240 mm, indicating that a slip reaching the failure of the concrete specimen was concentrated within this interval. However, no prominent rule was observed for the change in POM fiber content. As *c*/*d* increases, *s_u_* exhibits a monotonously rising trend, suggesting that the increase in *c*/*d* has a significant impact on *s_u_*.

The shape parameter *α* of the rising section is not strongly correlated with the POM fiber content, but it changes sharply, indicating that no evident relationship between the rigidity of the ascending portion of the bond–slip curve and the fiber content. Figure 8c shows that with the increase in *c*/*d*, *α* primarily exhibits a monotonously decreasing trend, which indicates that the growth in the concrete relative protective layer thickness has a great impact on the stiffness of the ascending portion of the bond–slip curve.

In summary, as *c*/*d* increases, *τ_u_* will increase, *s_u_* will monotonously increase, and *α* will decrease monotonously. According to different scholars, the shape parameter *α* of slip *s_u_* and the rise section corresponding to peak stress are affected by different factors, such as concrete strength, relative protective layer thickness, internal constraints of concrete, reinforcement stirrup, reinforcement form, pouring and curing conditions, and loading rate. The influence of POM fiber content on the slip *s_u_*, which is associated with the maximum stress and shape parameter *α* of the rising section, is not prominent. The test results are relatively discrete but exhibit a monotonously increasing or decreasing trend with the change in *c*/*d*.

#### 3.3.3. Influence of Different Anchorage Lengths

**Figure 9:**
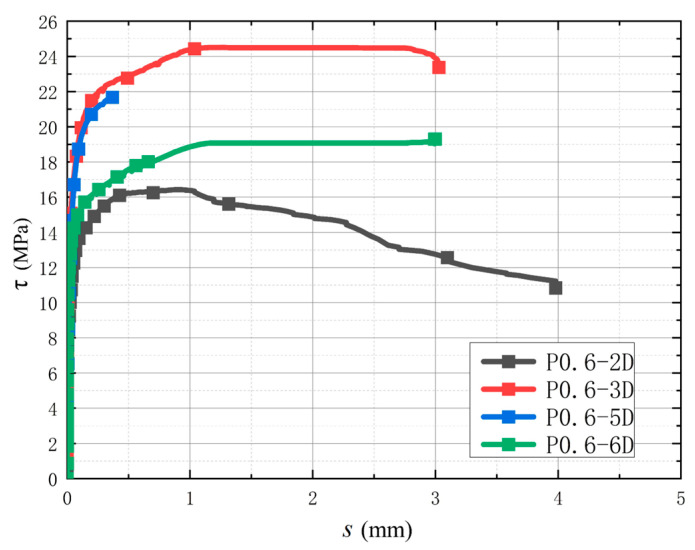
Bond–slip curves of POM0.6 specimens with different anchorage lengths.

As with ordinary reinforced concrete, when the reinforcement bonding length of CAC exceeds a certain value, the peak bond stress decreases significantly as bonding length rises. Figure 9 indicates that when the POM fiber content is 0.6 and the relative bonding length increases from 2 *d* to 3 *d*, the peak stress increases by approximately 49.33%. However, with the increase in the relative bond length from 3 *d* to 5 *d*, the peak value of stress decreases by approximately 11.58%. As it increased from 5 *d* to 6 *d*, the peak stress decreased by approximately 10.79%. The distribution of bond stress along the bonding length was uneven; for a shorter bonding length, the high-stress zone is longer, and the mean bond stress is larger. When the bonding length is longer, the high-stress zone is shorter, and the mean bond stress is smaller.

#### 3.3.4. Influence of Different Steel Bar Surface Morphologies

**Figure 10:**
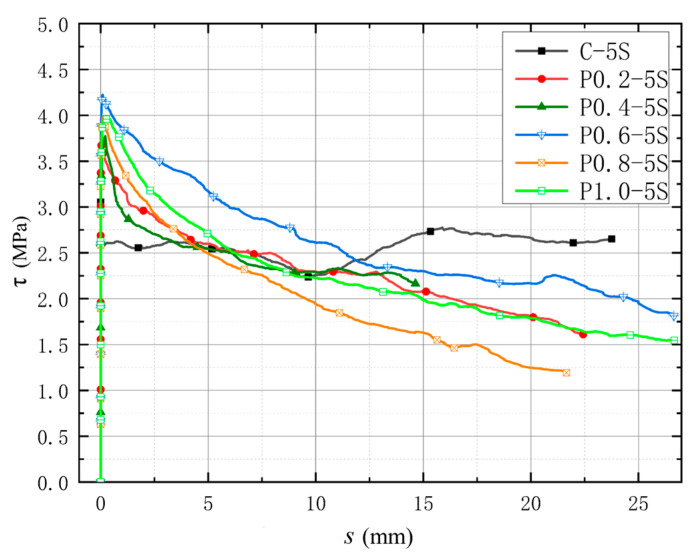
Bond–Slip Curve of Plain Round Reinforcement Bars under Central Pulling with Different POM Fiber Content.

Figure 10 shows the bond–slip curve between the circular reinforcement bar and CAC. Noticeably, a complete bond–slip curve can be obtained in the center drawing test for the light circular reinforcement bar, and the form of the curve remains unchanged. As the buckling loading was reached, the bond stress quickly increased, and the preslip of the peak bond stress of the bonded specimen was very small. After reaching the peak bond stress, the bond stress rapidly reduced to a certain value and then slowly increased. Unlike in the deformed reinforcement bar, the bonding force in the plain round reinforcement bar is derived from chemical bonding force and friction resistance. The chemical bonding force is lost and the stress reaches peak bond stress when the free end of the plain round steel bar has a small slip; therefore, the peak bond stress corresponds to a small slip. After reaching the peak bond stress, the bond stress increased due to the gradual accumulation of friction on the bond surface, and the bond stress increased with slip.

**Figure 11:**
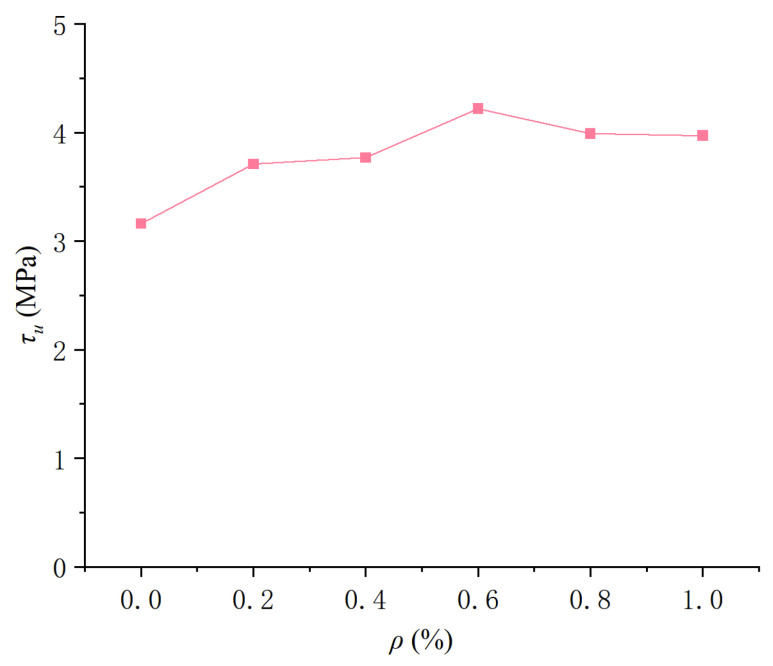
Peak bond stress of deformed and plain round steel bars under different treatment methods.

Figure 11 shows the variation in the peak bond stress of the round steel bar center drawing specimen with the POM fiber content. It indicates that the peak bond stress of the deformed reinforcement bar and round steel bar specimens had the same variation law with POM fiber content, and the peak bond stress first increases and then decreases as POM fiber content increases. The bonding strength of the deformed reinforcement bar and the round reinforcement bar increased by 32.60% and 33.54% compared to that of the CAC specimen, respectively, when the POM fiber content was 0.6. When other conditions were the same, the bond strength of the light circular reinforcement bar specimen was 19–21% that of the deformed reinforcement bar specimen.

## 4. Proposed and Verified Bond–Slip Constitutive Model

### 4.1. Proposed Model

From the aforementioned discussion and the corresponding analyses [38], the following model (Equations (4)–(6)) was developed, considering the ratio of the POM fiber content and reinforcement diameter to the protective layer of concrete (*c*/*d*), to represent the bond–slip behavior of the reinforcement and POM-fiber-reinforced CAC.

(4) $$\tau_{u}=\left\{\begin{array}{c} c/d=2,\;3,\;4.2\\ p_{1}\rho^{2}+p_{2}\rho +p_{3},\;0\leq \rho \leq 1.0 \end{array}\right.$$

(5) $$s_{u}=p_{4}\frac{c}{d}+p_{5}$$

(6) $$\alpha =p_{6}\frac{c}{d}+p_{7}$$

where *τ_u_* represents the maximum bond stress, *s* represents slip, *s_u_* represents the homologous slip to the peak bond stress, and *α* represents the form parameter of the rising portion of the bond–slip curve.

The above model parameters were obtained via nonlinear regression analysis in MATLAB based on the least square method using the test data. The specific results of the suggested model coefficients are presented in Table 12 and Table 13, and the curve characteristic values of the suggested bond–slip model of each deformed reinforcement bar and CAC for different *c*/*d* values are calculated. The bond–slip curve can be acquired by substituting the model in Section 3.2.

### 4.2. Model Verification

**Figure 12:**
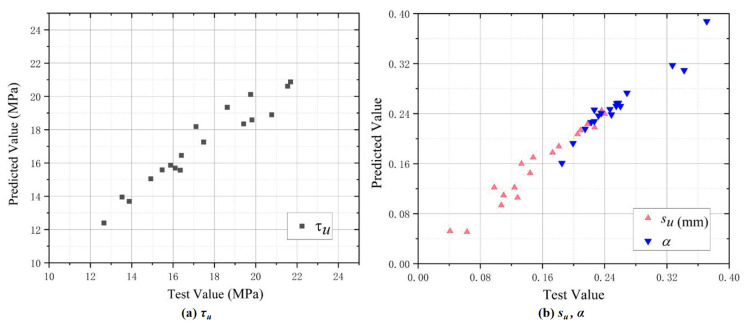
Verification of curve eigenvalue of the proposed model: (**a**) *τ_u_*; (**b**) *s_u_*, *α*.

**Figure 13:**
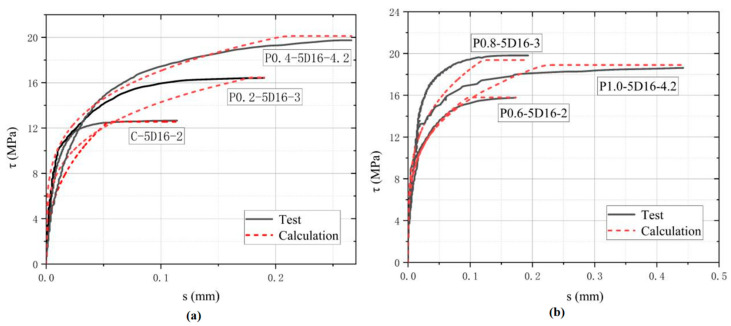
Comparison of the test results with the calculated results of the proposed bond–slip modell: (**a**) C-5D, POM0.2-5D, POM0.4-5D; (**b**) POM0.6-5D, POM0.8-5D, POM1.0-5D.

For model verification, the eigenvalues of the three curves calculated from Equations (4)–(6) were compared with the test results. Figure 12 indicates the eigenvalues obtained from the test data, which were taken as the horizontal coordinate. The predicted values calculated from Equations (4)–(6) were taken as the vertical coordinate, where the data points below the diagonal represent that the predicted values are lower than the test results. Anything above the diagonal is higher than the test result. Figure 12 shows that most of the data points were concentrated near the diagonal line, indicating that the consistency between the values calculated from the suggested bond–slip model and the test results is acceptable. In addition, six specimens, C-5D-2, POM0.2-5D-3, POM0.4-5D-4.2, POM0.6-5D-2, POM0.8-5D-3, and POM1.0-5D-4.2, were selected according to Equations (4)–(6) to calculate three curve characteristic values to plot the bond–slip curve. The latter was used to verify the applicability and accuracy of the suggested equations and determine the cumulative error of the bond–slip model and parameter calculation. Figure 13 illustrates the good fitting accuracy of the proposed bond–slip model in the first half of the ascent section, while the fitted values in the second half of the ascent section are smaller than the experimental values, and the peak stresses are basically similar to the fitted results in the parallel section.

## 5. Basic Anchorage Length Design Recommendations

Optimizing the basic anchorage length is the premise for ensuring that the reinforced CAC structure is not only economical but also in keeping with safety and reliability standards while giving full play to its bearing capacity. In the test study, when the influence of anchorage length was ignored, the distribution of bond stress along the reinforcement direction was generally considered uniform when the anchorage length *l_a_* ≤ 5 *d*. When the anchorage length *l_a_* ≥ 5 *d*, the bond stress increased with the reinforcement anchorage length, and the distribution became more uneven. If the bond stress is assumed to be equally distributed over the reinforcement length, then the bond stress will increase with the anchorage length. The minimal embedment length of the steel bar in concrete when the reinforcement drawing force is equivalent to the ultimate load is referred to as the basic anchorage length.

The anchorage length calculation formula in the provisions on the basic anchorage length of the reinforcement in Chinese standard GB/T50010-2010 [64] is shown in Equation (7). The standard also provides the relationship between axial tensile strength and cube compressive strength, as given in Equation (8). Table 14 presents the calculated basic anchorage length for 16 mm diameter HRB400 steel bars in CAC. These results can serve as reference data for engineering applications, with adjustments to be made based on actual construction conditions.

(7) $$l_{ab}=\alpha \frac{f_{y}}{f_{t}}d$$

(8) $$f_{t}=0.395f_{cu}^{\;0.55}$$

where *l_ab_* is the basic anchorage length of the tension reinforcement, *α* is the appearance coefficient of the anchorage reinforcement (0.14 for the deformed reinforcement), *f_y_* is the design value of the reinforcement tensile strength, *f_t_* is the tensile strength of the concrete axis, and *d* is the reinforcement diameter. 

## 6. Conclusions

In this study, the influences of fiber content, ratio of concrete protective layer thickness to reinforcement diameter *c*/*d*, bonding anchorage length, and surface shape of reinforcement bar on the bonding property of reinforcement and CAC were studied through a bonding test for reinforcement bar and POM-fiber-reinforced CAC. A 28 d bonding–slip model for deformed reinforcement bars and CAC was established, and the following conclusions were drawn:

(1) Split, pull-out with split, and pull-out failure are the bonding failure modes of deformed reinforcement bar and CAC. The thickness of the protective layer will affect the bonding failure mode. The thinner the protective barrier, the greater the likelihood of split failure. The bonding failure mode between the plain round steel bar and CAC is the pull-out failure mode. POM fiber has a small influence on the failure mode of the plain round reinforcement bar.
(2) The bond–slip buckling of the deformed reinforcement bar and POM-fiber-reinforced CAC is divided into three stages: microslip, slip, and parallel stages. For different fiber contents, as *c*/*d* increases, the peak bond stress increases, the steepness of the curve is greater, and the characteristic value *α* of the rising portion of the curve is smaller; therefore, the larger *c*/*d* is, the faster the bond stress rises within the same range.
(3) The peak bond stress *τ_u_* of the deformed reinforcement bar and POM-fiber-reinforced CAC initially rises and then subsequently declines as the POM fiber content increases, and the increase in the peak stress is the most prominent when the POM fiber content is 0.6. Compared to CAC without fiber, an increase of 49.69% can be achieved with a POM fiber content of 0.6. The shape parameter *α* of the slip *s_u_* and the rise section corresponding to the peak stress are concentrated in a certain interval, and the former has no strong correlation with the fiber content. However, with the change in *c*/*d*, *s_u_* increases monotonically, and *α* decreases monotonically.
(4) From the existing bond–slip curve calculation model, a 28 d bond–slip curve model for the deformed reinforcement bar and CAC was established considering POM fiber content and the ratio between concrete protective layer thickness and reinforcement diameter. This was then compared with the experimental results. The findings indicate that the model fits well with the experimental findings.
(5) The basic anchorage length of HRB400 reinforcement and CAC was calculated according to design codes, and the recommended basic anchorage length of each POM fiber content was obtained for reference in engineering applications.

In this study, a new type of fiber-reinforced CAC was developed using coral aggregates and POM fibers. Compared with ordinary CAC, its bonding performance with steel bars was significantly improved, demonstrating the potential of POM-fiber-reinforced CAC in sustainable marine engineering construction. The comprehensive investigation of its performance characteristics provides a foundation for understanding the macroscopic bond behavior of reinforced CAC. Further research will focus on the detailed characterization of its micro- and macrostructure, including the interfacial morphology and fiber dispersion mechanisms, to clarify the underlying bonding mechanism and provide deeper insight into its structure–property relationships.

## Figures and Tables

**Table 1:** Summary table of the effects of fiber types on concrete properties.

Fiber Type	Effects on Concrete Performance	Limitations/Drawbacks	Reference
Polyoxymethylene (POM)	Improves compressive, tensile, flexural strength, ductility and crack resistance, making the concrete less brittle and more durable under load	limit the durability of the concrete, and excessive fiber content can increase porosity or reduce strength	[43,44]
Polypropylene (PP)	Improves crack resistance, ductility, toughness, and bond strength; enhances durability	Poor dispersion, weak bond with cement matrix, reduced workability at high content	[45,46]
Polyvinyl Alcohol (PVA)	Increases tensile/flexural strength, ductility, and chemical bonding with matrix	High water absorption, workability loss, fiber clumping at high dosage	[47,48]
Polyethylene (PE)	Enhances tensile strength and ductility, good chemical resistance	Limited improvement in bond strength, may require surface treatment	[47,49]
Polyvinyl Chloride (PVC)	Increases compressive, flexural, and impact strength at optimal content; improves toughness	Workability decreases with higher content, performance drops above 1% fiber content	[50]
Glass Fiber	Improves tensile/flexural strength, bond strength, and durability	Susceptible to alkali attack, long-term durability concerns	[51,52]
Basalt Fiber	Increases compressive/flexural strength, impact resistance, and bond with matrix	Excessive content reduces ductility, higher cost	[53]

**Table 2:** Physical properties of POM fiber.

Density (kg/m^3^)	Tensile Strength (MPa)	Elongation (%)	Melting Point (°C)	Elastic Modulus (GPa)
1400	967	18	165	8

**Table 3:** Basic mechanical properties of reinforcement.

Diameter (mm)	Form	Yield Strength (MPa)	Ultimate Strength (MPa)	Elongation After Fracture (%)	Elastic Modulus (MPa)
16	deformed	416	622	28	2 × 10^5^
16	round	314	435	29	2 × 10^5^

**Table 4:** Parameter indicators of cement.

Specific Surface Area (m^2^/kg)	Standard Consistency (%)	Setting Time (min)		Compressive Strength (MPa)		Flexural Strength (MPa)		Loss on Ignition (%)
		Initial Set	Final Set	3 d	28 d	3 d	28 d	
360	28.00	225	295	33.6	55.7	6.3	8.6	1.80

**Table 5:** Parameter indicators of fly ash.

Fineness (%)	Water Demand Ratio (%)	Loss on Ignition (%)	Chlorine Ion Content (%)	Sulfur Trioxide (%)	Rate of Water Content (%)
12	95	5	0.02	5	0.1

**Table 6:** Parameter indicators of mineral powder.

Specific Surface Area (m^2^/kg)	Liquidity Ratio (%)	Loss on Ignition (%)	Chlorine Ion Content (%)	Sulfur Trioxide (%)	Rate of Water Content (%)
380	92	3	0.01	4	0.2

**Table 7:** Mix proportions of all specimens with and without POM fiber.

Types	Cement (kg/m^3^)	Coarse Aggregate (kg/m^3^)	Sand (kg/m^3^)	Water (kg/m^3^)	Mineral Powder (kg/m^3^)	Fly Ash (kg/m^3^)	POM Fiber (kg/m^3^)	Water Reducer (%)	W/B	Slump (mm)
CAC	330	620	1037	140	110	110	0	2	0.25	175
CAC-POM0.2	330	620	1037	140	110	110	2.8	2	0.25	113
CAC-POM0.4	330	620	1037	140	110	110	5.6	2	0.25	95
CAC-POM0.6	330	620	1037	140	110	110	8.4	2	0.25	87
CAC-POM0.8	330	620	1037	140	110	110	11.2	2	0.25	69
CAC-POM1.0	330	620	1037	140	110	110	14	2	0.25	55

**Table 8:** Results of mechanical tests of CAC with different mixes.

Types	Cube Compressive Strength (MPa)	Axial Compressive Strength (MPa)	Splitting Tensile Strength (MPa)
CAC	43.5	38.1	3.04
POM0.2	44.7	40.4	3.20
POM0.4	47.3	44.1	3.46
POM0.6	49.8	44.1	3.55
POM0.8	46.8	41.7	3.48
POM1.0	45.9	40.5	3.22

**Table 9:** Details of pulled-out test pieces.

No.	Fiber Content (%)	*d* (mm)	*la* (mm)	*c* (mm)	Steel Bar Surface Morphology
C-5D-2	-	16	80	32	D
C-5D-3	-	16	80	48	D
C-5D-4.2	-	16	80	67	D
POM0.2-5D-2	0.2	16	80	32	D
POM0.2-5D-3	0.2	16	80	48	D
POM0.2-5D-4.2	0.2	16	80	67	D
POM0.4-5D-2	0.4	16	80	32	D
POM0.4-5D-3	0.4	16	80	48	D
POM0.4-5D-4.2	0.4	16	80	67	D
POM0.6-5D-2	0.6	16	80	32	D
POM0.6-5D-3	0.6	16	80	48	D
POM0.6-5D-4.2	0.6	16	80	67	D
POM0.8-5D-2	0.8	16	80	32	D
POM0.8-5D-3	0.8	16	80	48	D
POM0.8-5D-4.2	0.8	16	80	67	D
POM1.0-5D-2	1.0	16	80	32	D
POM1.0-5D-3	1.0	16	80	48	D
POM1.0-5D-4.2	1.0	16	80	67	D
C-5S-4.2	-	16	80	67	S
POM0.6-5S-4.2	0.6	16	80	67	S
POM0.2-5S-4.2	0.2	16	80	67	S
POM0.4-5S-4.2	0.4	16	80	67	S
POM0.8-5S-4.2	0.8	16	80	67	S
POM1.0-5S-4.2	1.0	16	80	67	S
POM0.6-2D-4.2	0.6	16	32	67	D
POM0.6-3D-4.2	0.6	16	48	67	D
POM0.6-6D-4.2	0.6	16	96	67	D

**Table 10:** Pull-out test results.

No.	Average Value of Ultimate Load *F* (kN)	*τ_u_* (MPa)	*s_u_* (mm)	Failure Mode
C-5D-2	50.85	12.65	0.041	S
C-5D-3	55.75	13.87	0.128	S
C-5D-4.2	65.70	16.35	0.148	S
POM0.2-5D-2	54.62	13.53	0.124	S
POM0.2-5D-3	65.96	16.40	0.173	PS
POM0.2-5D-4.2	78.03	19.41	0.236	P
POM0.4-5D-2	59.99	14.92	0.063	PS
POM0.4-5D-3	70.03	17.11	0.098	PS
POM0.4-5D-4.2	80.62	19.75	0.205	P
POM0.6-5D -2	64.94	16.10	0.107	PS
POM0.6-5D-3	83.61	20.77	0.133	PS
POM0.6-5D-4.2	87.19	21.68	0.240	P
POM0.8-5D-2	64.14	15.88	0.110	PS
POM0.8-5D-3	79.63	19.81	0.144	PS
POM0.8-5D-4.2	86.77	21.54	0.181	PS
POM1.0-5D-2	58.63	15.47	0.209	PS
POM1.0-5D-3	77.55	17.48	0.227	PS
POM1.0-5D-4.2	84.3	18.62	0.218	PS
POM0.6-6D-4.2	93.26	19.34	1.119	PS
POM0.6-3D-4.2	59.12	24.52	1.017	PS
POM0.6-2D-4.2	26.4	16.42	0.892	P
C-5S-4.2	12.70	3.16	-	P
POM0.2-5S-4.2	14.93	3.71	-	P
POM0.4-5S-4.2	15.16	3.77	-	P
POM0.6-5S-4.2	16.98	4.22	-	P
POM0.8-5S-4.2	16.02	3.99	-	P
POM1.0-5S-4.2	15.96	3.97	-	P

Note: P denotes the pull-out failure mode, S indicates split failure mode, and PS represents pull-out with split failure mode.

**Table 11:** Characteristic value of bond–slip curve.

No.	*τ_u_* (MPa)	*s_u_* (mm)	*α*
C-5D-2	12.652	0.041	0.3712
C-5D-3	13.874	0.128	0.3421
C-5D-4.2	16.347	0.148	0.2147
POM0.2-5D-2	13.526	0.124	0.3271
POM0.2-5D-3	16.402	0.173	0.2266
POM0.2-5D-4.2	19.411	0.236	0.1849
POM0.4-5D-2	14.919	0.063	0.2686
POM0.4-5D-3	17.108	0.098	0.2602
POM0.4-5D-4.2	19.749	0.205	0.2225
POM0.6-5D-2	16.099	0.107	0.2555
POM0.6-5D-3	20.768	0.133	0.2547
POM0.6-5D-4.2	21.681	0.240	0.2467
POM0.8-5D-2	15.875	0.110	0.2577
POM0.8-5D-3	19.812	0.144	0.2266
POM0.8-5D-4.2	21.543	0.181	0.1992
POM1.0-5D-2	15.467	0.209	0.2319
POM1.0-5D-3	17.477	0.227	0.2486
POM1.0-5D-4.2	18.619	0.218	0.2356

**Table 12:** Calculation results for *p*_1_, *p*_2_, and *p*_3_ parameters of the proposed model.

Coefficient	*c*/*d* = 2	*c*/*d* = 3	*c*/*d* = 4.2
*p*_1_	−5.749	−12.78	−12.66
*p*_2_	8.935	16.34	16.44
*p*_3_	12.4	13.7	15.57

**Table 13:** Calculation results of *p*_4_, *p*_5_, *p*_6_, and *p*_7_ parameters of the proposed model.

Coefficient	CAC	POM0.2	POM0.4	POM0.6	POM0.8	POM1.0
*p*_4_	0.0535	0.0560	0.071	0.0665	0.0355	0.0045
*p*_5_	−0.0548	0.0097	−0.091	−0.0395	0.0385	0.2045
*p*_6_	−0.07825	−0.0711	−0.0213	−0.0044	−0.02925	0.0018
*p*_7_	0.5441	0.4595	0.3158	0.2655	0.3156	0.2332

**Table 14:** Calculation of recommended anchorage length.

Types	*f_cu_* (MPa)	*f_t_* (MPa)	*l_ab_*
CAC	43.5	3.15	16.02 *d*
POM0.2	44.7	3.19	15.78 *d*
POM0.4	47.3	3.29	15.30 *d*
POM0.6	49.8	3.39	14.87 *d*
POM0.8	46.8	3.28	15.39 *d*
POM1.0	45.9	3.24	15.55 *d*

## Data Availability

The original contributions presented in this study are included in the article. Further inquiries can be directed to the corresponding author(s).
